# An Immunological Glance on Pancreatic Ductal Adenocarcinoma

**DOI:** 10.3390/ijms21093345

**Published:** 2020-05-08

**Authors:** Michael Karl Melzer, Frank Arnold, Katja Stifter, Friedemann Zengerling, Ninel Azoitei, Thomas Seufferlein, Christian Bolenz, Alexander Kleger

**Affiliations:** 1 Department of Urology, Ulm University Hospital, 89081 Ulm, Germany; michael.melzer@uniklinik-ulm.de (M.K.M.); Friedemann.Zengerling@uniklinik-ulm.de (F.Z.); Christian.Bolenz@uniklinik-ulm.de (C.B.); 2 Department of Internal Medicine I, Ulm University Hospital, 89081 Ulm, Germany; frank.arnold@uni-ulm.de (F.A.); katja.stifter@uni-ulm.de (K.S.); ninel.azoitei@uni-ulm.de (N.A.); Thomas.Seufferlein@uniklinik-ulm.de (T.S.)

**Keywords:** PDAC, immunotherapy, pancreatic cancer, immune checkpoints, immune microenvironment

## Abstract

Pancreatic ductal adenocarcinoma (PDAC) has still a dismal prognosis. Different factors such as mutational landscape, intra- and intertumoral heterogeneity, stroma, and immune cells impact carcinogenesis of PDAC associated with an immunosuppressive microenvironment. Different cell types with partly opposing roles contribute to this milieu. In recent years, immunotherapeutic approaches, including checkpoint inhibitors, were favored to treat cancers, albeit not every cancer entity exhibited benefits in a similar way. Indeed, immunotherapies rendered little success in pancreatic cancer. In this review, we describe the communication between the immune system and pancreatic cancer cells and propose some rationale why immunotherapies may fail in the context of pancreatic cancer. Moreover, we delineate putative strategies to sensitize PDAC towards immunological therapeutics and highlight the potential of targeting neoantigens.

## 1. Introduction

Pancreatic cancer is predicted to be the second leading cause of cancer-related death until 2030 [1]. Up to date, patients suffering from pancreatic ductal adenocarcinoma (PDAC), the most frequent type of pancreatic cancer, have a five-year overall survival rate of less than 9% [2]. This can be attributed to the late diagnosis paralleled by the difficulty of complete tumor resection and early metastases in 80 to 90% of the cases [2]. Poor prognosis is also the result of ineffective treatment strategies through chemotherapy resistance [3,4]. Current first-line treatment strategies in unresectable PDAC include monotherapies such as gemcitabine or 5-fluorouracil (5-FU) and combination therapies such as gemcitabine plus nab-paclitaxel or FOLFIRINOX, (5-FU, folinic acid, irinotecan, and oxaliplatin) in metastatic pancreatic cancer [3]. However, conventional chemotherapeutic treatment only leads to modest clinical outcomes for PDAC patients. Targeting subsets of PDAC patients based on their mutational profile, for example, with defective DNA-damage response, poly (ADP-ribose) polymerase 1 (PARP)-inhibitors, may represent a potential alternative. The treatment of patients featuring germline breast cancer susceptibility protein (BRCA) mutations with PARP-inhibitor olaparib, as maintenance therapy, led so far to a significant improvement in progression-free survival of pretreated patients but did not improve overall survival in these patients [5]. Although different genetic mutational patterns lead to various subtypes of PDAC, the overall breakthrough for mutagenome-based treatment strategies is still pending [4].

Other innovative therapeutic approaches in oncology involve immune checkpoint inhibitors (ICIs) such as the most prominent ipilimumab (cytotoxic T-lymphocyte-associated protein-4 (CTLA-4) inhibitor) or nivolumab (programmed cell death protein-1 (PD-1) inhibitor). The intervention with these inhibitors for the treatment of unresectable or metastatic melanoma was approved by the Food and Drug Administration (FDA) in 2011 and 2014, respectively. In the meanwhile, a variety of other immune checkpoint inhibitors (such as atezolizumab, avelumab, pembrolizumab, etc.) and other indications for the application of ICIs have been approved. Of note, the PD-1 blocker pembrolizumab has been approved by the FDA for the treatment of solid cancers with high microsatellite instability (MSI-H) or deficient mismatch repair (dMMR) including PDAC in 2017 [6,7]. Despite the broad application of immune checkpoint inhibitors and other immunotherapeutic approaches, for example, adoptive T cell therapy [8] or peptide vaccination, approaches [9] for PDAC have failed to translate into a meaningful improvement in the overall survival for the majority of PDAC patients. Despite the unfortunate outcome of immunotherapeutic treatment strategies in the context of PDAC, Balachandran et al. identified a robust neoantigen-specific T cell response against PDAC in long-term survivors of PDAC [10]. This showed that at least some PDACs are, per se, able to activate effector T cells as one reason to potentially control tumor growth.

To date, it appears that both standard chemotherapeutic and immunotherapeutic regimens have very limited efficacy in patients suffering from PDAC. Since, in a subset of patients, the innate immune system appears to be more effective against PDACs than conventional therapeutics, we focused in the current review on the immune cell landscape of these tumors. The interaction and communication between different immune cells with tumor and stromal cells were given special significance. Potential targets and translational models of PDACs’ immune microenvironment were also discussed. Lastly, application of ICI and potential hurdles in a PDAC setting and their prone-to-fail mechanisms, as well as future perspective and immunotherapeutic tools that may enhance the response to ICI therapy, were addressed. A discussion about alternative treatment strategies in the context of immunotherapy will give a comprehensive conclusion of this review.

## 2. Immunologic Subtypes of PDAC

**Figure 1:**
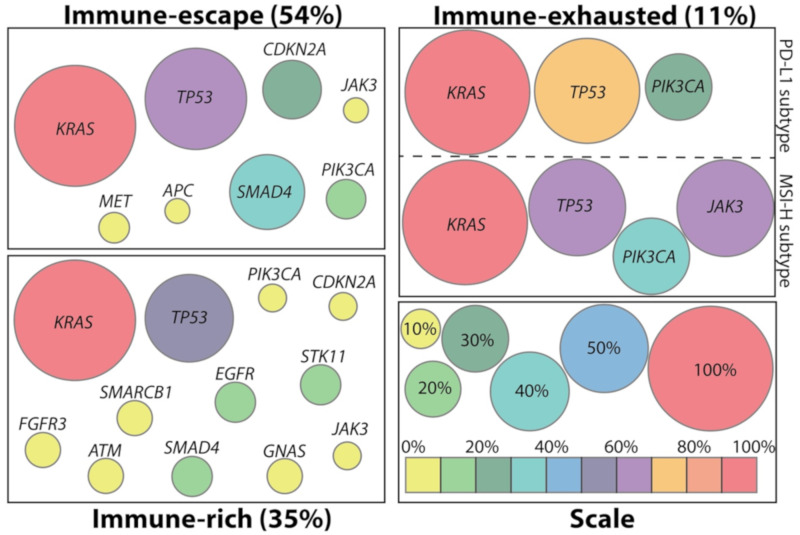
Mutational pattern in different immune-phenotypes of pancreatic ductal adenocarcinoma (PDAC). The mutational pattern of human PDAC defines three subtypes: Immune-escape, immune-rich, and immune-exhausted. Circle size is indicative of the frequency of mutations in the indicated gene. Circle size was calculated based on data from Wartenberg et al. [14]. A scale for circle size, color, and mutation frequency is given on the right bottom panel.

Genetic mutations mainly drive the initiation and progression of PDAC. Usually, a PDAC patient harbors, on average, 32 genetic mutations within approximately 10 signalling pathways [11]. The progression is characterized by metaplasia of acinar cells to ductal cells, followed by the development of three different kinds of nonmalignant precursor lesions, namely: (1) Pancreatic intraepithelial neoplasm (PanIN), (2) intraductal papillary mucinous neoplasm (IPMN), and (3) mucinous cystic neoplasm (MCN) [4]. Accumulation of key driver mutations such as the activation of *KRAS* in 92% of the cases, paralleled by the disruption of cell cycle checkpoint genes, like *TP53*, *CDKN2A*, or *TP53BP2*, in 78% of all human pancreatic cancers finally leads to an invasive PDAC [11]. Aside from the common mutations, genes involved in DNA-repair, such as *BRCA1*, *BRCA2*, *PALB2*, and *ATM,* though less frequently mutated [11], contribute to a more aggressive phenotype of PDAC and not only influence survival but also treatment outcome [12,13]. Despite the contribution of specific mutations to the progression and aggressiveness of PDAC, the genetic landscape may be less relevant for chemotherapeutic resistance [4]. Instead, the response to chemotherapy appears to be defined by the phenotype of PDAC [4]. Nevertheless, the genetic landscape defines the immune environment in human PDAC. Wartenberg et al., correlated immune cell infiltration with the corresponding mutational pattern of human PDAC and distinguished three PDAC subtypes concerning their immunogenicity (Figure 1) [14]. The authors investigated resected human pancreata of patients with stage I to III PDAC. Tumors of patients that were treated neoadjuvantly or that reached stage IV were not included in the analysis [14].

The primary subtype found in 54% of PDACs reflected an *immune-escape* subtype. This is characterized by low infiltration of T and B cells, displays high infiltration with regulatory T cells (Tregs), is associated with a high *KRAS*, *TP53*, *CDKN2A*, *SMAD4*, and *PIK3CA* and low *ERBB4* and *MET* mutational burden, and has a poor outcome with a median overall survival (OS) of around 10 months. By contrast, the second subtype, called immune-rich and observed in 35% of all PDAC cases, displayed high infiltration of T and B cells and low infiltration of Tregs. It was associated with a lower mutational burden than the previous group, concerning *CDKN2A*, *SMAD4*, and *PIK3CA*, and showed a median overall survival of 19 months. As the only subtype, this group includes mutations in *GNAS* and *IDH2*. Forty-four percent of all immune-rich patients have been attributed the most favorable outcome from the analyzed cohort showing a median overall survival of 23 months. This subgroup was characterized by an additional high frequency of *STK11, ATM*, and *SMARCB1* mutations. The less frequent subtype is the immune-exhausted group. Only 11% showed an immune-exhausted phenotype, with a median OS of 10 months, comparable to that of the *immune-escape* subtype. Within this subtype, two subgroups could be identified. One was associated with high programmed cell death 1 ligand 1 (PD-L1) expression, high CD8^+^/regulatory T cell (Treg) ratios, and with a high mutational burden in *PIK3CA*. The second subgroup that features microsatellite instability and loss of DNA mismatch repair genes has the highest CD8^+^/Treg ratios and, beside *PIK3CA* mutations, has also frequent mutations in *JAK3*. [14]

Although the study correlated the immune phenotypes of PDAC with overall survival rates, it did not specify the approached therapeutic regimes. We, therefore, cannot exclude differences in the treatment of various subgroups of patients with potential impact on the OS. Since tumors were classified retrospectively, this study also leaves room for speculation with regard to the possibility for all patients to have undergone state-of-the-art therapy at the specific time point during their treatment. Taken together, based on the mutational pattern that is present within the tumors it becomes evident that, aside from common mutations as in the *KRAS* and *TP53* gene, distinct mutational changes define the immune phenotyping of PDAC. Of note, the immune-rich phenotype displays the most diverse genetically altered subgroup in this study [14]. The significance of those findings might rely on a potential benefit from an ICI therapy for those patients that harbor many mutations and display the immune-rich phenotype. The ideal prerequisite of an abundance of inflammatory and tumoricidal immune cells for ICI therapy is regarded in the immune-rich phenotype. Of note, the immune-exhausted phenotype with the MSI-H subgroup displays features for which PD-1/PD-L1 blockade is justified. We found essential for the current review that the demonstration of correlation between specific mutational patterns of PDAC and certain immune phenotypes should be substantiated by an explanation of how different cell types interact and influence the shaping of the immune milieu.

## 3. Potential Cell Types Involved in Disease Pathogenesis

**Figure 2:**
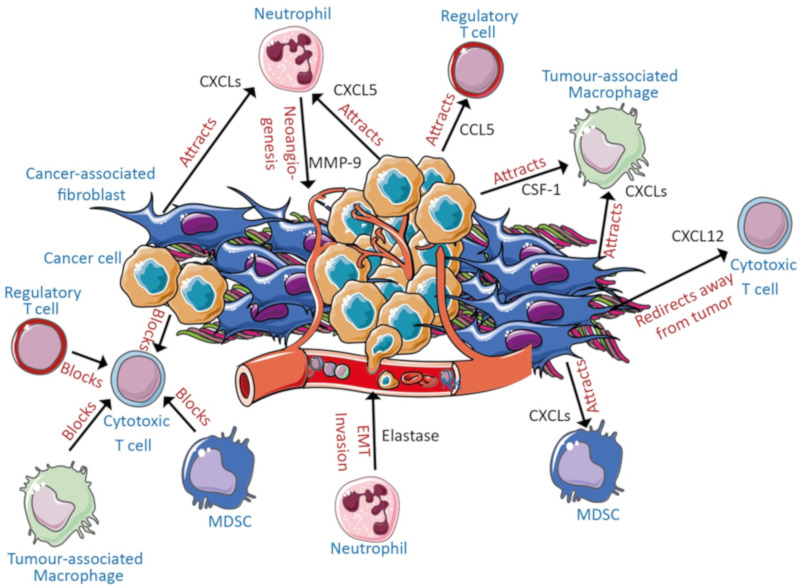
Major contributors to the immune microenvironment in PDAC. Cancer cells and cancer-associated fibroblasts (CAFs) secrete different chemokines and factors to attract immunosuppressive immune cells or block the expansion of cytotoxic T cells in PDAC.

The different cell types within pancreatic cancer shape a specific immune-environment, which contributes to the pathogenesis of PDAC (Figure 2).

### 3.1. Cancer Cells and Stroma

Cancer cells secrete chemokine (C-C motif) ligand 5 (CCL5) to attract immunosuppressive Tregs [15], C-X-C motif chemokine 5 (CXCL5) to recruit neutrophils [16] and colony-stimulating factor-1 (CSF-1) to attract macrophages [17]. Further, cancer cell-derived exosomes lead to the expansion of immature myeloid cells [18] and expansion of both M1 pro-inflammatory macrophages and alternatively activated M2 immunosuppressive macrophages [19] in vitro. Cancer cells are also able to inhibit cytotoxic T cell functions by secretion of transforming growth factor β (TGF-β) [20]. Within this intricate network of signals, cancer cells render cytotoxic T cells anergic while simultaneously generating a highly immunosuppressive environment which further maximizes the inhibitory effects on tumoricidal cells.

Tumor cells are surrounded by a dense stroma containing cancer-associated fibroblasts and different immune cells. Cancer associated fibroblasts (CAFs) are part of the stroma and can arise from pancreatic stellate cells (PSCs) and also contribute to an immunosuppressive microenvironment [21]. CAFs and activated PSCs, which represent one of the primary sources for generating stroma, induce neoangiogenesis by promoting the secretion of vascular endothelial growth factor (VEGF) [22]. CAFs and PSCs also facilitate the process of invasion and metastasis by inducing the epithelial-to-mesenchymal (EMT) program in the cancer cells [22,23,24,25]. By secretion of TGF-β, CAFs can induce a double positive phenotype in PDAC cells, which is defined by proliferation as well as the induction of an EMT phenotype [26]. Further, activated PSCs can attract CD8^+^ T cells by secretion of CXCL12, which targets the C-X-C chemokine receptor 4 (CXCR4), thereby preventing them from reaching tumor cells as shown in a murine model [27]. Furthermore, cancer cells have the ability to trigger CAFs to secrete CXCLs, shown to attract myeloid-derived suppressor cells (MDSC), M2 tumor-associated macrophages (TAMs), and neutrophils [28].

To date, three different kinds of cancer-associated fibroblasts were described in pancreatic cancer (inflammatory CAFs (iCAF), antigen-presenting CAFs (apCAF), and myofibroblastic CAFs (myCAF)), which mostly render the tumor microenvironment a benefiting landscape for tumor progression [29,30]. The iCAFs, located a certain distance from tumor cells, promote an inflammatory environment through the activation of interleukin-1 (IL-1)/JAK-STAT3-signalling pathway [30,31]. The iCAF-secreted interleukin (IL)-6 [30] promotes an immunosuppressive environment (defined by few infiltrating effector T cells and high expression of PD-L1) and a more invasive PDAC phenotype triggered by STAT3 activation [32,33]. In parallel, iCAF-secreted macrophage colony stimulated factor (M-CSF) was reported to drive the polarization of M2 macrophages, which additionally favors tumor progression and invasiveness [34]. The apCAFs, on the other hand, can present antigens to T cells [29]. However, in this context, due to a lack of co-stimulatory molecules, T cells are not able to proliferate, suggesting an immunosuppressive role for apCAFs by decreasing the CD8^+^ to Treg ratio [29]. Both iCAFs and apCAFs are currently considered to favor tumor progression [29]. In contrast, depletion of myCAFs leads to increased numbers of intratumoral Tregs and higher degrees of dedifferentiation and invasion [35], indicating an essential role in preventing the accumulation of immunosuppressive, pro-tumorigenic cells and pro-invasive phenotype. This appears to conflict with the role of iCAFs promoting an immunosuppressive environment, as mentioned above. It becomes evident that the influence of CAFs on metastasis is highly dependent on the predominating CAF population.

Knudsen and colleagues identified three different types of stroma (mature, intermediate, and immature) based on the content of CAFs and presence of collagen fiber and loose stromal matrix [36]. According to their classification, immature stroma with less collagen and high numbers of CAFs were associated with modest survival (median survival 281 days), which was also observed by Miksch et al. [36,37]. By contrast, mature stroma, which consisted of few CAFs and dense collagen, showed the best survival (median survival 1033 days) in this study [36]. On the other hand, intermediate stroma, a mixture of the mature and immature subtypes, correlated with moderate survival (median survival 656 days) [36]. In another study, large amounts of intratumoral collagen and intratumoral CD3^+^ tumor-infiltrating lymphocytes (TILs) were associated with improved survival [37]. Mahajan and colleagues confirmed in resected human PDACs that the stroma subtype significantly influences patient survival [38]. Accumulation of a fibrolytic stroma (defined as stroma with high-smooth muscle actin α-smooth muscle actin (α-SMA) and low collagen content was associated with substantially reduced survival, and it was characterized by high infiltration of immunosuppressive CD206^+^ M2 macrophages and low infiltration of CD8^+^ T cells as well as CD68^+^ M1 macrophages [38]. On the other hand, fibrogenic stroma (defined as stroma with high α-SMA and collagen expression) was associated with increased progression-free survival (PFS) and was paralleled by higher infiltration of CD8^+^ T cells and CD68^+^ M1 macrophages than in fibrolytic stroma [38]. The overall survival of PDAC patients highly depends on the type of stroma and potentially also on the stromal plasticity. Steins and colleagues demonstrated that cancer cells with epithelial phenotype could activate PSCs and thus lead to the deposition of collagen [39]. In contrast, mesenchymal cancer cells featuring elevated secretion of CSF-1 show opposite effects [39], indicating that stromal composition and its plasticity may be reflected by the differentiation grade of cancer cells.

The tumor stroma is a major component of PDAC. It shapes the tumor microenvironment and modulates the composition of the immune cell landscape, which is further paralleled by a high impact of different stromal subtypes on the overall survival. In the following, we will focus on the immune and stromal cells contributing to the immunosuppressive microenvironment before apprehending the detailed role of T cells in the context of PDAC.

### 3.2. Myeloid-Derived Suppressor Cells, Neutrophils, and Macrophages

Myeloid-derived suppressor cells (MDSCs) exert mostly immunosuppressive functions within PDAC. They suppress T cell function and efficiently enhance metastasis [40,41]. Neutrophils seem to act mostly as promoters of tumor progression as indicated by the induction of EMT and angiogenesis after the secretion and release of matrix metalloproteinase-9 (MMP-9) and elastase [42,43,44,45]. The pro-inflammatory M1 and immunosuppressive M2 macrophages [46] mostly act pro-tumorigenically in PDAC by inducing neoangiogenesis, EMT, and metastasis [19,34,47]. This is rather intriguing when considering a variety of other cancer entities (such as lung cancer or ovarian cancer) where pro-inflammatory M1 macrophages mostly act as a tumor suppressor translating to better prognosis [48,49]. During PDAC inflammation, M1 macrophages rather favor the initiation of acinar-to-ductal metaplasia (ADM) [50,51]. Oncogenic KRAS mutations in acinar cells translate to an augmented attraction of inflammatory macrophages which will then support the progression of acinar cells to PanINs [51].

### 3.3. T Cells

The analysis of PDAC patients and healthy control individuals revealed that systemically prevalent CD4^+^ T cells and CD14^+^ macrophages are the most drastically altered immune cells in PDAC [52]. In macrophages 261 genes (involved in the cell cycle, inflammation, coagulation, adhesion and development) were significantly altered, whereas 496 genes (involved mostly in apoptosis, cell cycle, inflammation, and DNA-damage) were differently expressed in CD4^+^ T cells [52]. A higher systemic number of PD-1^+^ CD4^+^ T cells was observed in PDAC patients when compared to the healthy controls [52]. PD-1^+^ and FoxP3^+^ CD4^+^ T cells infiltrated PDAC at high rates [52]. Collectively, these data demonstrate that CD4^+^ T cells represent one of the primary immune cell populations that are affected by PDAC. Activated CD4^+^ effector T cells and Tregs were reported to support EMT of (pre-)malignant pancreatic cells upon the secretion of IL-6 and tumor necrosis factor (TNF) α in vitro [53], indicating the tumor-promoting role of CD4^+^ T cells. However, to that, Wang and colleagues correlated high infiltration of CD4^+^ T cells to rather a better survival of PDAC patients [54]. The immunosuppressive features of the Tregs among tumor-infiltrating CD4^+^ T cells in human PDAC were acknowledged by several other studies [27,55,56,57]. While the infiltration of especially Tregs and M2 macrophages increases during carcinogenesis of PDAC, the number of CD8^+^ T cells substantially decreases [58,59]. This indicates that Tregs support the progression of PDAC. Indeed, Kenkel and colleagues did demonstrate that Tregs facilitate the development of liver metastasis in murine PDAC [60]. Taken together, it becomes evident that Tregs are tumor-promoting, whereas the role of the effector CD4^+^ T cells is debatable.

CD8^+^ T cells and natural killer (NK) cells represent two major immune cell populations that have the capability, though with moderate success, to negatively impact the progression of the tumor. Cytotoxic CD8^+^ T cells, for example, are in general less abundant in human PDACs compared to other cancer entities such as melanoma [61]. Sakellariou-Thompson et al., found less than 1% of CD3^+^ tumor-infiltrating leukocytes (TILs) of total cells in resected human PDACs while detecting around 2% of CD3^+^ TILs in human melanoma samples [61]. This was associated with fewer CD8^+^ T cells than CD4^+^ T cells, indicated by a comparably low CD8:CD4 ratio of 0.75, supported as well by other studies [27,61]. As a comparison, highly immunogenic melanomas [62] showed a CD8:CD4 ratio of 1.5 in the study of Sakellariou-Thompson et al. [61]. Altogether, these studies indicate that PDAC remains poorly infiltrated by tumoricidal effector CD8^+^ T cells. Interestingly, Helm and colleagues demonstrated that a higher degree of dedifferentiation (G3 tumor vs. G1 and G2 tumors) was paralleled by a decreased number of CD8^+^ T cells within human tumor samples [55]. Furthermore, the size of the lesions negatively correlated with CD8^+^ T cell infiltration and function [63]. Smaller metastatic lesions were corroborated with higher infiltration of the activated, non-exhausted CD69^+^ PD-1^-^ CD8^+^ T cells, whereas bigger cancerous lesions harbored mainly an exhausted CD69^-^ PD-1^+^ CD8^+^ T cell subpopulation [63]. This implies that CD8^+^ T cells are either excluded from dedifferentiated and large tumors or show no ability to inhibit progression and dedifferentiation of tumors. However, not only the CD8^+^ T cells’ local infiltration seems to be diminished in PDAC patients, but also their systemic distribution in the peripheral blood [64,65]. Both local and systemic reduced numbers of CD8^+^ T cells were associated with poor survival of PDAC patients [27,64]. Inactivation of CD8^+^ T cells was mainly triggered by PD-L1/PD-1 interaction between tumor cells and T cells or between T cells and dendritic cells (DCs) [33,66]. In this context, DCs show upregulation of PD-L1 and further secretion of immunosuppressive TGF-β and IL-10 in murine systems [33,66]. Murine DCs further suppress CD8^+^ T cells by the PD-L2/PD-1 interaction and simultaneously induce expansion of Tregs by CD80 and induced costimulatory ligand(ICOSL) signalling [60]. In light of these immunosuppressive mechanisms leading to the inactivation of CD8^+^ T cells, ICI therapy might render as one of the most suitable strategies for targeting CD8^+^ T cells.

### 3.4. NK Cells

Natural killer (NK) cells are generally described to inhibit tumor growth. NK cells and their role in preventing relapse after pancreatectomy [67] and metastasis [68] can be mimicked in mouse models, thus offering clues about potential post-pancreatectomy premisses. However, their killing capacity may be hindered by a plethora of signalling molecules (such as TGF-β 1 derived from exosomes, or soluble UL16 binding protein) that promote the escape of cancer cells from the recognition by NK cells [57,68,69,70]. Intriguingly, PDAC patients harbor a high number of circulating NK cells (CD56^+^ CD3^−^) in the peripheral blood [57]. However, the penetration of these cells into human PDAC tissue is rather low, which may be traced back to a lack of proper chemokine signalling for the recruitment of circulating NK cells to the tumor [57]. The number of NK cells is not decreased in PDAC patients, like in the case of CD8^+^ T cells, yet they do not have the ability to reach the tumor, a fact that may elucidate the involvement of various mechanisms contributing to the antitumor immune response.

### 3.5. B Cells and Mast Cells

Another cell population, which defines the immune cell landscape in PDAC, is represented by the B cells. Depending on their intratumoral distribution, B cells can either exert pro-tumorigenic and immunosuppressive functions as single dispersed B cells or show tumor-suppressive and immunostimulatory effects when organized as lymphoid structures [71,72]. Although less well known for their function in tumors, mast cells seem to support tumor growth by conferring resistance to chemotherapy while supporting invasion and migration of cancer cells [73], enhancing proliferation of PDAC and pancreatic stellate cells [74].

Taken together, cancer cells and different kinds of immune cells cooperate and communicate to form a mostly tumor-friendly environment. The largest fraction is comprised of tumor-promoting populations such as CAFs, Tregs, MDSCs, and TAMs, while CD8^+^ T cells and NK cells with tumor-repressing functions are in the minority.

## 4. Translational Models to Study PDAC-Immune System Interactions

Investigating PDAC in patients is often limited to a mere description of the tumor microenvironment. To circumvent this, we sought to address various state-of-the-art model systems.

The use of innovative cell culture systems allows mimicking the detailed interaction of (at least) two different cell populations. For example, one common in vitro setting is the retrieval of cell culture supernatants (also called conditioned medium), rich in various factors from the “donor” population, to overlay onto and stimulate a “receiver/target” cell population. Herein, it was demonstrated that exosomes secreted by cancer cells induce expansion of immature myeloid cells [18], M1 and M2 macrophages [19], while inhibiting the function of NK cells [69,70]. These results might explain how the cancer cells contribute to the immune environment that is observed in patients. An alternative possibility of studying communication between various cell populations involves co-culture experiments, for example, dendritic cells and peripheral blood mononuclear cells (PBMCs) in alloreactive stimulation assays [65,75]. Taken together, innovative cell (co-)culture models may allow the investigation of the two-way influence of molecules secreted by cancer cells and immune cells but also the direct cell-cell interactions in order to gain mechanistic insight about a particular tumor. To substantiate the in vitro culture models, the use of rodent model systems is mandatory.

Among the most prominent PDAC model systems is the LSL-Kras^G12D/+^, Trp53^fl/+^, Pdx1-Cre (KPC) mouse model, which was first described in 2005 [76]. An excellent overview of the KPC mouse model involving activation of the murine oncogenic *Kras* and conditional expression of a dominant-negative form of *Trp53* in the pancreas was described by Lee and colleagues [77]. The significant advantage of this model lies in the recapitulation of the human PDAC setting by showing similar clinical and histopathological features [77]. An alternative to genetically engineered mouse models is the use of allo-/ xenograft mouse models [78] that offer the possibility to transplant murine as well as human tumor cells hetero- or orthotopically into the (most often immunodeficient) mice [78]. This allows the in vivo investigation of individual tumors from patients with a particular mutational pattern [78]. In contrast to transgenic mouse models, xenograft models need less time for investigations and are less cost intensive [78].

The KPC and other mouse models allow investigating the interaction of the stroma compartment and the immune system [27,79]. For example, these mouse models enable the characterization and validation of PD-1/PD-L1, PD-L2 immune checkpoint inhibition, and the interaction of CD8^+^ T cells with cancer cells or dendritic cells [33,60,66]. Tregs and their influence on metastatic development can also be monitored in mouse models [52]. While aware of the limitations associated with murine model systems, the current involvement of engineered mice or xenograft approaches offer crucial knowledge to understanding the microenvironment, initiation, progression, metastasis, and immune suppression in PDAC [77]. However, these models cannot fully recapitulate the in vivo situation in humans.

In the last years, the development of organoid cultures started, at least partially, to compensate for the shortcomings of the mouse models. Organoids allow the investigation of the murine and human PDAC as well as non-neoplastic pancreatic structures while mimicking the cellular interactions of the original tumor [80]. The employment of various extracellular matrix formulations allows cancer cells to acquire and develop into three-dimensional structures [80]. Apart from recapitulating the original features of PDACs, organoids were rendered suitable for performing drug screenings with good predictability both in patients and mice [80,81]. Furthermore, co-culture of pancreatic cancer-derived organoids and T cells accurately rehearse the complex microenvironment of PDAC [82]. Tsai and colleagues demonstrated that T cells added to PDAC organoid cultures invaded the matrigel and accumulated near tumor cells [82]. All these studies favor the further employment of the organoids for the investigation of PDAC microenvironment, drug response, and biological behavior of tumor cells.

## 5. Clinical Application of Immune Checkpoint Inhibitors and Generation of Neoantigens in PDAC

### 5.1. Application of Immune Checkpoint Inhibitors in PDAC

The following section is dedicated to the treatment of PDAC involving immune checkpoint inhibitors targeting the CTLA-4 and PD-1 axis. Upon inhibition of either of the two immune checkpoint-signalling pathways, only a modest prolongation of overall survival was achieved, which will be presented in the following section. However, the potential success of ICIs may rely on the pre-existing T cell receptor (TCR) repertoires and combination with other (immunotherapeutic) drugs. So far, no breakthrough has been reported after treating PDAC patients with immune checkpoint inhibitors. At the time of preparation of this manuscript, only one phase 3 study involving ICIs in the PDAC setting in the database of clinicaltrials.gov was registered (Table 1).

Targeting the CTLA-4 molecule on Tregs is expected to unleash the cytotoxic T cell response against tumors. The effectiveness of CTLA-4 inhibition by ipilimumab was compared to the combined treatment with ipilimumab and GVAX (an irradiated allogeneic pancreatic cancer cell-based granulocyte-macrophage colony-stimulating factor (GM-CSF) vaccine) in 30 patients with advanced disease after gemcitabine-based chemotherapy in a phase 1b study [83]. This study revealed that the median overall survival of 4.2 months did not significantly differ between the two treatment arms [83]. However, a slightly better prognosis was attributed to patients subjected to combined drug intervention [83]. Taken together, targeting the CTLA-4 axis only led to a modest therapeutic response as the survival of 4.2 months is extremely short, indicating this intervention in PDAC patients is rather inefficient. Interestingly, the treatment of PDAC patients with ipilimumab or nivolumab (PD-1 inhibitor) led to a diversification of their T cell repertoire, which can be enhanced by the addition of GVAX [84]. In this particular setting, the combination of GVAX and ipilimumab appears more efficient in inducing the diversification of T cell repertoire than the combination with nivolumab [84]. This might render the CTLA-4 blockade as more critical than PD-1 blockade for the establishment of a broad antitumor immune response in PDAC. Concordant with this hypothesis, direct benefit for patients was evident for the ipilimumab-mediated induction of the broad diversification of T cell receptor repertoire [84]. To note, a strong effect of ipilimumab in broadening the immune response relays on individual patient’s characteristics. Thus, patients displaying high diversity of TCR clonality before treatment followed by high proliferation of specific clones after ipilimumab therapy were associated with better survival than patients having a more unfavorable TCR repertoire at baseline (median survival 8.66 months vs. 4.28 months) [84]. Another phase 1b study involving the combination of ipilimumab and gemcitabine concluded an overall survival of 6.9 months for 21 patients with an advanced (previously pretreated) disease with a tolerable toxicity profile [85]. Furthermore, patients benefited from combined therapeutic approaches as the additive effect of GVAX, PD-1 inhibitors, or chemotherapeutic drugs became evident. An important point is that a prerequisite for successful therapy is given by the presence and the diversity of tumor-reactive T cells. Involving the ICI therapy for patients with that prerequisite will most likely help in amplifying and unleashing the pre-existing antitumor immunity, especially in the context of CTLA-4 blockade.

In the following, we would like to address the PD-1/PD-L1 axis. Eleven PDAC patients still exhibiting progression under the first-line therapy exhibited an observable response to the combination therapy with pembrolizumab (a PD-1 blocker) and the oncolytic reovirus pelareorep, resulting in median overall survival of 3.1 months [86]. Although this therapeutic approach leads to a measurable response in PDAC, it becomes evident again that the therapy only leads to a short extension of survival. Again, the analysis of the T cell receptor clonality in this study revealed the most extended survival for one patient, which was corroborated with the induction of the highest number of new clonal T cells following the combination therapy [86]. This highlights the dependency of PD-1 blockade success in PDAC on pre-existing patients’ TCR repertoire. As mentioned above, pembrolizumab was approved for the treatment of solid tumors with dMMR and MSI-H, including PDAC [6,7]. Despite this promising ratification, it has to be mentioned that especially PDAC only harbors few cases with MSI-H or dMMR [87]. Interestingly, those subjects with dMMR or MSI-H seem to represent a group of PDAC patients with prolonged overall survival compared to the generality of PDAC patients [88,89]. This fact might potentially be attributed to an elevated mutational burden, neoantigens, and higher immunogenicity [87]. In line with poor survival extension after ICI therapy, the KEYNOTE-158 study recruited 233 patients with noncolorectal cancer featuring MSI-H/dMMR [90]. Among these patients, 22 PDAC patients (mostly with previous therapy) had a median overall survival of 4.0 months after therapy with pembrolizumab [90]. Of note, PDAC and brain tumors, which were among the seven most prevalent cancer entities involved in this study, recorded the worst response rate with 18.2% and 0% response rate and poorest overall survival rates of 4.0 and 5.6 months, respectively [90]. Interestingly, the survival curves of other tumor entities, such as endometrial or gastric cancers, did not even reach the point to determine a median overall survival this study [90], indicating that MSI-H/dMMR in solid malignancies are, per se, well targetable by pembrolizumab. This study indicates that even PDAC with genomic instability and anticipated elevated amounts of neoantigens is rather difficult to be targeted by PD-1 blockade. Another study investigating pembrolizumab in different entities of cancers with 24 PDAC patients documented an overall survival of 3.9 months [91]. The analysis of the mutational burden revealed that in 16 out of 20 investigated tumor entities with the highest mutational burden showed the best response to the treatment with pembrolizumab [91].

Since PD-1 blockade is tantamount to little or no success, one could reason that targeting PD-1/PD-L1 axis by PD-L1 blockers may also be prone to fail. Testing of combined durvalumab (PD-L1 blocker) and tremelimumab (CTLA-4 blocker) interventions versus durvalumab monotherapy delineated only a poor response rate of 3.1% and 0%, respectively, in a pretest cohort of 65 patients with metastatic PDAC, thus preventing the study from being extended to a larger cohort [92]. Similarly, treatment of stage III/IV PDAC with durvalumab and ibrutinib (Bruton tyrosine kinase inhibitor) revealed a poor response rate of 2% and 4.2 months median overall survival [93]. Taken together, these studies indicate that also targeting PD-L1 does not improve the prognosis of PDAC patients.

Altogether, the intervention with immune checkpoint inhibitors, alone, combined, or in a cocktail with other therapeutic agents, only leads to a poor improvement of overall survival. Interestingly, however, there seems to be a correlation between the appearance of a broad polyclonal T cell response, the presence of high mutational burden in tumors, and the clinical outcome. Of note, patients benefit the most from immune checkpoint blockade if they have a broad TCR repertoire before the beginning of therapy. An overview of patients in the cited studies, therapeutic approaches, and outcomes is presented in Table 2.

### 5.2. Neoantigens in PDAC

Balachandran et al., delineated the differences between the very few long-term survivors and the other PDAC patients [10]. They could demonstrate that long-term survivors (more than six years for 82 patients) exhibited a significantly higher number of neoantigen reactive and TCR repertoire diverse CD8^+^ T cells, which were thought to maintain the tumors with a median of 38 neoantigens per tumor under control [10]. Interestingly, during the metastatic progression of the PDAC, long-term survivors, the number and quality of especially Mucin-16 (MUC16) neoantigens were diminished again [10]. Mature dendritic cells, Tregs, and macrophages belonged to the cell populations found elevated in the tumor immune cell landscape of long-term survivors [10]. 221 PDAC patients were screened for potentially targetable neoantigens and showed 4 to 4000 potential neoantigens per tumor [94]. Of note, the number of identified neoantigens was much lower than in melanoma tumors (11 to 14,000), which can be excellently targeted by immunotherapies [94]. In particular, DC-, peptide-, or messenger ribonucleic acid (mRNA)-based multispecific neoepitope vaccines were successfully tested in melanoma to induce tumor-specific T cell responses, providing early evidence of antitumor activity in patients with solid tumors [95,96,97]. Excellent reviews about the recent advances in adoptive cell therapy and neoantigens-based vaccines are given by Peng et al., as well as Ott and colleagues [98,99].

Knudsen et al., further showed that the highest neoantigen load was associated with those tumors which arose from microsatellite unstable background [36]. Although antigens can be presented in PDAC, the activation of cytotoxic CD8^+^ T cells remains absent [94]. Further, a correlation between high mutational load in PDAC and a low number of present cytotoxic CD8^+^ T cells was observed [94]. ICI therapy might provide one approach to activate the non-activated tumor-reactive T cells.

Overall, the clustering of PDAC based on the expression of neoantigens, stromal composition, and infiltration of immune cells revealed four subtypes [36]. Interestingly the “cold” cluster with few neoantigens, immune infiltrations, and mutational load (aside from *KRAS*) showed the longest survival [36]. On the other hand, the “hot” cluster with high numbers of all immune cells and mutational burden and the “mutationally active” cluster with many mutations and infiltration of TILs and peritumoral lymphocytes, as well as few infiltrating macrophages, showed significantly poorer survival than the “cold” cluster [36]. Potential explanations for those seemingly contradictory observations might be that “cold” tumors harbor quite a few mutations (except for *KRAS*) and show mature stroma indicating a more differentiated tumor onset. On the other hand, the “hot” and “mutationally active” tumors have high expression levels of immune checkpoint inhibition molecules and macrophages [36]. The study suggests that immune checkpoint inhibition in “hot” and “mutationally active” tumors might be limited by the nontargeting effect of immunosuppressive macrophages [36]. Taken together, PDAC presents neoantigens, though to a much lower extent than in melanoma, one of the best targetable tumors by immune checkpoint inhibition. Although those neoantigens might be targeted by endogenous T cells, their efficacy is limited due to immunosuppressive mechanisms, which cannot be entirely reversed by immune checkpoint inhibition as other mechanisms (for example, macrophages) or yet unknown cellular or molecular events are likely to be involved in this suppression.

## 6. Conclusions, Outlook, and Future Perspectives

Immune checkpoint blockade in the context of PDAC seems to be less effective than in other cancer entities. The main reason appears to be the poor immunogenicity of PDAC, which challenges the activation of the host immune system to clear cancer. A potential new option for potentiating the outcome of immune therapy against PDAC is represented by the targeting of neoantigens within the frame of combination therapies. Generating neoantigens by inducing inflammation, for example, by oncolytic viruses or cell-based vaccination approaches, is promising, yet to date not sufficient. Further “education” of the immune system toward these neoantigens is needed to achieve a promising killing impact on tumor cells. This can be envisaged by immune checkpoint blockade, which unravels mostly T cells from the anergic state, though a more sophisticated and more broadened approach is needed to circumvent the highly immunosuppressive immune microenvironment in PDAC.

Adoptive cell therapy (ACT) with genetically engineered cells might provide an alternative for treatment in PDAC. Most recently, chimeric antigen receptor (CAR) T cells have gained clinical approval for the treatment of CD19^+^ B cell malignancies [100], which supports the feasibility and success of this therapeutic regime. In the context of PDAC, the situation is far more complicated due to the highly immunosuppressive microenvironment and the dense stroma, which poses a difficulty for the T cells to penetrate and reach the cancer cells [101]. A recent review by Fan and colleagues offers an insight into the ongoing clinical trials involving ACT for the treatment of PDAC [102]. A plethora of studies using ACT is indicative of hope for future developments involving specific cell therapies for PDAC. However, these are only some little first steps before potential clinical approval of adoptive cell therapy for this deadly disease.

Other approaches aiming at treatment of PDAC from an immunological site encompass the application of vaccines. Peptide and DNA, as well as cell-based (DC), are currently under clinical investigation and show some moderate success [103]. A combination of GVAX vaccine with immune checkpoint inhibitors [83,84] was briefly presented in this manuscript.

In addition, the application of bispecific T cell engagers (BiTEs), which avoids the necessity of inducing or engineering antigen-specific T cells might surface as another option in PDAC treatment. This is performed through binding of tumor antigens and simultaneous activation of T cells by BiTEs. An already successful clinical application of BiTEs conducted in CD19^+^ B cell lymphomas [104] may render this approach as promising. However, the highly immunosuppressive environment of PDAC with T cells dampened in their function by a considerable amount of antagonists poses broad application of BiTEs under incertitude.

Taken together, a plethora of immunotherapies have been designed in recent years in oncology. Many studies are currently investigating the feasibility and efficacy of immunotherapies in the context of PDAC, but, so far, no breakthrough has been achieved in the context of pancreatic cancer. For future developments, favoring combinatorial approaches involving several different immunotherapeutics might represent a reasonable option with a better chance for considerable success in PDAC treatment.

## Figures and Tables

**Table 1:** Phase-3 clinical trial with application of approved immune checkpoint inhibitors for the treatment of PDAC.

ICI Class	Drugs	Patients/Disease	Treatment	Clinical Trial	Status
PD-1 blocker, CTLA-4 blocker	Pembrolizumab and/or ipilimumab	Patients with hepatocarcinoma, lung cancer, melanoma, renal cancer, head and neck cancer, pancreatic cancer, ovarian cancer, colorectal cancer, cervical cancer, breast cancer	Vein, artery, or intra-tumour infusion of checkpoint inhibitor	NCT03755739	Recruiting

Clinicaltrials.gov was searched for the following combinations and selection criteria: Selection: “Phase 3”, combinations of “pancreatic cancer” or “PDAC” and “ipilimumab”, “tremelimumab”, “nivolumab”, “pembrolizumab”, “durvalumab”, “avelumab”, “atezolizumab”, or “cemiplimab”. Pancreatic cancer is highlighted to enable a more rapid recognition of the relevant disease concering this review.

**Table 2:** Immune checkpoint inhibitor application in PDAC patients.

ICI Class	Co-Drugs	Treatment	Patients/Disease	Median Overall Survival	Reference
CTLA-4	GVAX	Ipilimumab ± GVAX	Previously treated, advanced PDAC (*n* = 15 for iplimumab; *n* = 15 for ipilimumab ± GVAX)	Ipilimumab: 3.6 months; Ipilimumab ± GVAX: 5.7 months	[83]
CTLA-4, PD-1	GVAX, Listeria monocytogenes	Ipilimumab ± GVAX; GVAX+ mesothelin expressing Listeria monocytogenes± nivolumab	Metastatic PDAC (*n* = 25 for ipilimumab ± GVAX; *n* = 32 for GVAX+ mesothelin expressing Listeria monocytogenes± nivolumab)	n.a. for specific therapy	[84]
CTLA-4	Gemcitabine	Ipilimumab + gemcitabine	Advanced or metastatic PDAC (*n* = 21)	6.9 months	[85]
PD-1	Oncolytic virus (pelareorep), chemotherapy (gemcitabine or irinotecan or leucovorin and 5-FU followed by 5-FU	Pembrolizumab + pelareorep + chemotherapy	Advanced PDAC (*n* = 11)	3.1 months	[86]
PD-1		Pembrolizumab	Previously treated, advanced non-colorectal cancer with DNA mismatch repair or high microsatellite instability (*n* = 233; pancreatic cancer *n* = 22)	4.0 months	[90]
PD-1		Pembrolizumab	PD-L1 positive advanced solid tumors (*n* = 475; pancreatic cancer *n* = 24)	3.9 months	[91]
PD-L1 CTLA-4		Durvalumab ± temelimumab	Metastatic pancreatic cancer (*n* = 65)	n.a.	[92]
PD-L1	Bruton tyrosine kinase inhibitor ibrutinib	Ibrutinib + durvalumab	Previously treated, advanced PDAC, breast cancer, non-small cell lung cancer (*n* = 122, pancreatic cancer *n* = 49)	4.2 months	[93]

A scheme for the in text-mentioned studies is presented in this table. Class of immune checkpoint inhibitor (ICI) is given. Co-drugs are listed. Abbreviations are used as the following: CTLA-4, cytotoxic T-lymphocyte-associated protein 4; PD-1, programmed cell death protein 1; PD-L1, programmed cell death protein 1 ligand 1; GVAX, cell-based vaccine consisting of irradiated pancreatic cancer cells expressing a granulocyte-macrophage colony-stimulating factor (GM-CSF); n.a., not applicable.

## Abbreviations

5-FU	5-fluoruracil
ACT	adoptive cell therapy
ADM	acinar-to-ductal metaplasia
apCAF	antigen presenting CAF
BiTE	bispecific T cell engager
BRCA	breast cancer susceptibility protein
CAF	cancer associated fibroblast
CAR	chimeric antigen receptor
CCL	Chemokine (C-C motif) ligand
CSF-1	colony-stimulating factor-1
CTLA-4	cytotoxic T-lymphocyte-associated protein 4
CXCL	C-X-C motif binding chemokine
CXCR	C-X-C motif chemokine receptor
DC	dendritic cell
dMMR	deficient mismatch repair
EMT	epithelial to mesenchymal transition
FDA	Food and Drug Administration
FOLFIRINOX	5-fluoruracil, folinic acid, irinotecan, oxaliplatin
GM-CSF	granulocyte-macrophage colony-stimulating factor
iCAF	inflammatory CAF
ICI	immune checkpoint inhibitor
ICOSL	induced costimulatory ligand
IL	interleukin
IPMN	intraductal papillary mucinous neoplasm
M-CSF	macrophage colony-stimulating factor
MCN	mucinous cystic neoplasm
MDSC	myeloid-derived suppressor cell
MMP-9	matrix metalloproteinase-9
MUC16	Mucin-16
mRNA	messenger ribonucleic acid
MSI-H	high microsatellite instability
myCAF	myofibroblastic CAF
n.a.	not applicable
NK cell	natural killer cell
OS	overall survival
PanIN	pancreatic intraepithelial neoplasm
PARP	poly(ADP-ribose)-polymerase
PBMC	peripheral blood mononuclear cell
PD-1	programmed cell death protein 1
PD-L1	programmed cell death 1 ligand 1
PDAC	pancreatic ductal adenocarcinoma
PFS	progression-free survival
TAM	tumor-associated macrophage
TCR	T cell receptor
TGF-β	transforming growth factor β
TIL	tumor-infiltrating leucocyte
TNF-α	tumor necrosis factor α
Treg	regulatory T cell
α-SMA	α smooth muscle actin
